# Establishment of Sertoli Cell Lines from Giant Pandas (*Ailuropoda melanoleuca*)

**DOI:** 10.3390/ani16193132

**Published:** 2026-10-06

**Authors:** Lin Shi, Yuliang Liu, Rong Hou, Yi Zheng, Junhui An

**Affiliations:** 1 The Conservation of Endangered Wildlife Key Laboratory of Sichuan Province, Chengdu 610081, China; 2 Key Laboratory for Animal Genetics, Breeding and Reproduction of Shaanxi Province, College of Animal Science and Technology, Northwest A&F University, Yangling 712100, China; 3 Chengdu Research Base of Giant Panda Breeding, Chengdu 610081, China

**Keywords:** spermatogenesis, sertoli cell, giant panda, immortalization, testis

## Abstract

Giant pandas (*Ailuropoda melanoleuca*) are a critically protected endangered species in China, but current understanding of their testicular physiology and regulatory mechanisms for spermatogenesis remains limited. Sertoli cells (SCs), the sole somatic cell type within seminiferous tubules, play essential roles in nutritional support, blood-testis barrier (BTB) formation, and hormonal regulation, indispensable for germ cell development in testes. However, primary cultured SCs are prone to undergo replicative senescence, which severely impedes their in-depth studies in vitro. In this study, we introduced the SV40 large T antigen into giant panda primary testicular somatic cells via lentiviral transduction and then performed monoclonal isolation, eventually successfully establishing two immortalized cell lines (PD-SCs-1 and PD-SCs-2). These two cell lines exhibited long-term proliferative capacity and have been cultured for more than eight months and passaged over 70 times. They also stably expressed typical SC markers and retained intercellular junction-like structures and molecular responses to hormonal stimulation, supporting the preservation of several important SC characteristics. Yet, immortalization was accompanied by alterations in cellular properties, including reduced androgen receptor expression and chromosomal abnormalities. Together, the established PD-SCs-1 and PD-SCs-2 cell lines provide a valuable in vitro platform for in-depth investigation of the testicular niche, the mechanisms for spermatogenesis, and the pathology of reproductive disorders in giant pandas, and also lay the experimental groundwork for development of novel breeding technology and conservation of this endangered species.

## Simple Summary

Giant pandas (*Ailuropoda melanoleuca*) are an endangered species with limited knowledge of spermatogenesis and testicular development. As the only somatic cell type inside seminiferous tubules in favor of germ cell development, Sertoli cells rapidly cease proliferation and undergo senescence under standard culture conditions, hindering their long-term investigation in vitro. To address this issue, we introduced the SV40 large T antigen into giant panda primary testicular cells and successfully established two monoclonal Sertoli cell lines with long-term proliferative capacity. These cells have been maintained in continuous culture for over eight months while preserving several characteristic features of normal Sertoli cells. Although immortalization induced certain cellular alterations, these cell lines provide a valuable resource for studies on giant panda reproductive biology and lend support to conservation efforts.

## 1. Introduction

The principal physiological function of the male reproductive system is to support spermatogenesis, a highly coordinated biological process that begins with the self-renewal and differentiation of spermatogonial stem cells (SSCs). This is followed by a series of complex developmental events, including meiotic division of spermatocytes and spermiogenesis of spermatids, ultimately giving rise to functionally mature sperm. Spermatogenesis is indispensable for species propagation and the maintenance of genetic diversity [1,2]. Within the seminiferous tubules of the testis, spermatogenesis is tightly regulated by multiple testicular cell types. Among these, Sertoli cells (SCs), as a key somatic cell population, are essential for the maintenance of testicular homeostasis, and their functional integrity critically determines the efficiency and fidelity of spermatogenesis [3].

SCs possess unique morphological features and multiple physiological functions. As epithelial-like irregular polygon cells, SCs extend extensive cytoplasmic processes that interface with adjacent SCs and developing germ cells, providing the structural scaffold for epithelial intercellular junctions within the seminiferous tubules [4,5]. Functionally, SCs are primarily responsible for the formation of the blood-testis barrier (BTB), essential for maintenance of normal spermatogenesis. Situated in the basal compartment of the seminiferous tubules, the BTB divides the seminiferous epithelium into basal and adluminal compartments, and supports the orderly progression of meiosis [6,7,8]. Structurally, the BTB is composed of tight junctions, specialized extracellular matrix (ECM) structures, desmosomes, and gap junctions. Tight junctions between adjacent SCs constitute the core structural element, preserving BTB integrity and testicular niche homeostasis [3,9]. Tight junction-associated proteins, including zonula occludens-1 (ZO-1) and claudin 11 (CLDN11), are synthesized and secreted by SCs and play pivotal roles in regulation of the BTB function [10,11]. Moreover, SCs secrete a variety of growth factors, such as bone morphogenetic protein 4 (BMP4), stem cell factor (SCF), and glial cell line-derived neurotrophic factor (GDNF), coordinating the self-renewal, differentiation, and apoptosis of SSCs [12,13]. Disruptions in SC performance or population size derail the spermatogenic cycle, often culminating in fertility impairment marked by defective sperm production [14].

Primary SCs have a limited lifespan and low passage capacity in vitro and are prone to undergo senescence and functional deterioration, which markedly constrains their application to long-term and reproducible experimental studies. Hence, SC immortalization becomes an effective means in reproductive biology research [15]. To date, by using different technical strategies, including transfection with viral oncogenes such as the SV40 large T antigen, and transfection with the human telomerase reverse transcriptase (hTERT) gene, several SC lines from humans and rodents have been successfully established [1,15,16,17]. These immortalized cell lines generally preserve the morphological and phenotypic characteristics of primary SCs, stably express SC markers such as SRY-box transcription factor 9 (SOX9) and Wilms tumor protein 1 (WT1), and secrete spermatogenesis-related growth factors such as GDNF and SCF, with certain cell lines possessing both infinite proliferative capacity and the ability to exhibit BTB-like properties in vitro [18]. Indeed, immortalized SC lines represent powerful in vitro tools for studies on reproductive biology across multiple species. For instance, pharmacological intervention and genetic manipulation can be applied to SC lines to dissect hormone- and stress-mediated ferroptotic injury as well as hormone-responsive pathways in vitro, as demonstrated by recent studies that provided experimental evidence for exploration of intervention targets for testicular injury, thus suitable for in vitro mechanistic validation related to reproductive toxicity [19,20]. Like their rodent counterparts, immortalized SC lines from livestock including pigs and sheep also retain partial hormone-responsive capacity, and are mostly adopted to dissect cellular immunity and basic cellular functions in vitro, holding application potential for virus and drug vaccine screening [21,22]. Arguably, immortalized fish SC lines can be used to unravel signaling pathways underlying fish spermatogenesis, serving as reliable in vitro models for investigation of fish reproductive and developmental mechanisms [23]. As for human-derived immortalized SC lines, they feature stable passaging while maintaining the phenotypes and functions of primary cells, and can be employed to explore molecular mechanisms underlying male infertility induced by environmental pollutants, thereby constituting robust in vitro models for studies on human reproductive injury [17,24]. Despite all these, immortalized SC lines from rare and endangered species, including giant pandas, have so far not been reported.

Giant pandas (*Ailuropoda melanoleuca*) are a rare species exclusive to China and serve as a flagship species for global biodiversity conservation. Notably, captive male giant pandas often exhibit suboptimal reproductive performance, with approximately 30% of adult males showing variable degrees of reproductive dysfunction, which substantially limits the expansion of captive populations [25,26]. Accumulating evidence indicates that deletions and mutations in key reproduction-related genes, such as *DACH2*, *SYT6*, and *IQCD*, are important contributors to the low reproductive efficiency of giant pandas [27]. In addition, the testicular cell landscape and regulatory pathways of spermatogenesis in this endangered species remain inadequately understood. Such limited insights can directly be ascribed to scarce tissue access as well as the lack of robust in vitro platforms. Recently, single-cell RNA sequencing (scRNA-seq) technology was used to systematically characterize the cellular landscape of giant panda testes, leading to the identification of eight testicular cell subtypes, including SCs. Immunohistochemical analysis further validated the specific nuclear localization of WT1 in giant panda SCs [28], supporting its utility as a specific biomarker for this cell type. In addition, an immortalized peritubular myoid cell line derived from giant panda testes, designated PD-TPTCs, has recently been established [29], demonstrating the technical feasibility of immortalizing giant panda testicular somatic cells. Nevertheless, an immortalized SC line derived from giant pandas has not yet been reported.

We hypothesized that immortalization mediated by the SV40 large T antigen could also generate giant panda cell lines that preserve the intrinsic properties of SCs. This study hence aimed to establish and characterize SC lines from giant pandas. To this end, we introduced the SV40 large T antigen into giant panda primary testicular somatic cells via lentiviral transduction and then performed monoclonal isolation, eventually establishing two immortalized cell lines that were thoroughly characterized to verify their SC identity and functional integrity. Therefore, the successfully established giant panda SC lines provide a stable and abundant cellular reservoir for future functional investigation, potentially facilitating development of novel breeding technology and conservation of this endangered species.

## 2. Materials and Methods

### 2.1. Animals

Testicular samples used in this study were obtained post-mortem from a giant panda neonate. The neonate was born at the Chengdu Research Base of Giant Panda Breeding and died shortly after birth in October 2020 due to dysplasia. Post-mortem necropsy was performed by veterinarians following standard protocols. Testicular tissue samples were collected during necropsy for subsequent experimental research, and no morphological abnormality was observed in the testicular tissue. All animal procedures were in accordance with and approved by the institutional animal ethical committee of Northwest A&F University (Approval Code: DK-20200339; Approval date: 17 October 2020) and the relevant administrative department in Chengdu Research Base of Giant Panda Breeding.

### 2.2. Isolation of SC-Enriched Testicular Somatic Cells

Testicular tissues were minced and dissociated with 1 mg/mL Type IV collagenase (17104019; Thermo Fisher Scientific, Waltham, MA, USA) at 37 °C for 30 min. Tissues were further incubated in a mixture of 0.25% trypsin-EDTA (Hyclone, Logan, UT, USA) and 1 mg/mL DNase I (Thermo Fisher Scientific, Waltham, MA, USA) at 37 °C for 10 min to prepare a single-cell suspension of seminiferous tubules. Fetal bovine serum (FBS; Hyclone, Logan, UT, USA) was added to terminate enzymatic dissociation. The cell suspension was filtered through a 40 μm cell strainer (BD Biosciences, Franklin Lakes, NJ, USA) and centrifuged at 600× *g* for 10 min. The resultant SC-enriched pellet was collected, aliquoted, and cryopreserved for further experimental use.

### 2.3. Cell Culture

The cryopreserved primary testicular somatic cells were thawed and cultured according to standard protocols. Specifically, both primary and immortalized testicular SCs were cultured in the complete medium consisting of high-glucose DMEM (10-013-CVRC, Corning, NY, USA), 5% FBS (FBS-CE500, Newzerum, Christchurch, New Zealand), 1 × GlutaMAX (35050061, Thermo Fisher Scientific, Waltham, MA, USA), 1 × non-essential amino acids (11140050, Thermo Fisher Scientific, Waltham, MA, USA), 1 × penicillin-streptomycin solution (15140122, Thermo Fisher Scientific, Waltham, MA, USA), and 1 × Mycoplasma Elimination Reagent (40607ES08, Yeasen Biotechnology, Shanghai, China). Cells were maintained in a humidified incubator at 37 °C with 5% CO_2_. The medium was changed routinely, and cells were subcultured at a ratio of 1: 3 when reaching 80% of confluence to sustain favorable growth.

### 2.4. Immortalization of Giant Panda Testicular Somatic Cells

Cell immortalization was conducted following a previously established protocol [30]. Briefly, the transfer vector plox-Ttag-iresTK (Addgene No. 12246) encoding the SV40 large T antigen was co-transfected into HEK293T cells with the second-generation packaging vectors psPAX2 and pMD2.G. The harvested lentiviruses were concentrated by ultracentrifugation. Primary testicular somatic cells at passage 3 were seeded into 24-well plates for lentiviral transduction, with 10 μg/mL polybrene (HY-112735; MCE, Princeton, NJ, USA) added to the transduction medium. For spinfection, cells were subjected to centrifugation at 32 °C and 3000× *g* for 1 h, then maintained at 37 °C. Cells were refreshed with the complete medium 16 h after transduction.

### 2.5. Monoclonal Isolation

Immortalized testicular somatic cells at passage 5 were harvested and resuspended in the complete medium at a density of 5–10 cells/mL, then seeded into 96-well plates. Cell growth in each well was monitored daily under a microscope, and wells with a single cell were marked and recorded. When cells in marked wells reached approximately 80% of confluence, monoclonal colonies were dissociated using 0.25% trypsin-EDTA (Hyclone, Logan, UT, USA). Cells were then transferred to 24-well plates for expansion to generate sufficient monoclonal cells for subsequent experiments.

### 2.6. Cell Immunofluorescence

Cells were first seeded into 96-well plates. After attachment, cells were fixed with 4% PFA at room temperature for 10 min and permeabilized with 0.25% Triton X-100 for 15 min. Cells were then blocked with 3% bovine serum albumin (BSA; 08R81068-CF, MP Biomedicals, Santa Ana, CA, USA) at 4 °C for 2 h. After blocking, cells were incubated with the corresponding primary antibodies overnight at 4 °C, and detailed information of all primary antibodies is listed in Table 1. For the negative control, PBS replaced the primary antibody while all other procedures remained unchanged to rule out non-specific binding. After overnight incubation, primary antibody solution was removed, and cells were rinsed with PBS. Cells were then incubated with the Alexa Fluor 488-conjugated donkey anti-rabbit secondary antibody (1:400; Yeasen Biotechnology, Shanghai, China) at room temperature for 2 h. After incubation, cells were washed and counterstained with DAPI solution (1:1000; SL7100, Coolaber, Beijing, China) for 5 min at room temperature. Fluorescence images were acquired using a Nikon Eclipse 80i fluorescence microscope. For quantification, three independent biological experiments were performed for each group. In each independent experiment, three replicate wells were set as technical replicates for each condition, and a total of 200 cells were randomly selected and analyzed per group to calculate the positive cell rate.

### 2.7. Cell Proliferation Assay by CCK-8

Cell proliferation was evaluated using a Cell Counting Kit-8 (CCK-8; Mirus Biological, Xi’an, China) assay, in accordance with the manufacturer’s protocol. Briefly, primary testicular somatic cells and the derived monoclonal SC lines were seeded into 96-well plates at 2000 cells per well. At 0, 24, 48, and 72 h post-seeding, 10 μL of CCK-8 reagent were added to each well, followed by incubation at 37 °C for 4 h. Cell-free wells supplemented with the culture medium and CCK-8 solution were used as blank controls. The absorbance value at 450 nm was determined using a microplate reader. Growth curves were plotted based on the measured absorbance values to evaluate cell proliferative capacity. All CCK-8 assays were performed with three independent biological experiments. Five replicate wells were set for each group as technical replicates in each independent experiment, and the mean values of technical replicates from each experiment were used for subsequent statistical analysis. Two-way ANOVA was used for multi-group comparisons, followed by the Dunnett’s post hoc test to compare each experimental group against the control group at each time point.

### 2.8. EdU Staining for Cell Proliferation Analysis

Cell proliferative activity was evaluated using the BeyoClick^™^ EdU Cell Proliferation Kit with Alexa Fluor 488 (C0071S, Beyotime Biotechnology, Shanghai, China) according to the manufacturer’s instruction. Briefly, cells were seeded into 24-well plates and cultured overnight to allow complete adherence. Subsequently, the cells were incubated with the EdU working solution for 2 h to label newly synthesized DNA in proliferating cells. After incubation, the culture medium was discarded, and the cells were gently rinsed with PBS. The cells were then fixed at room temperature and permeabilized to facilitate reagent penetration. The click reaction solution was prepared and applied to the cells for 30 min at room temperature in the dark, followed by thorough washing to remove unbound dye. Cell nuclei were counterstained with the Hoechst solution under dark conditions. Fluorescence images were captured using a fluorescence microscope. The numbers of EdU^+^ and total cells were quantified via ImageJ 1.50b, with a minimum of 200 cells counted per group and three independent biological experiments performed for each group. The cell proliferation rate was calculated to determine the proliferative capacity of cells.

### 2.9. Western Blot Analysis

Total cellular proteins were extracted with RIPA lysis buffer (DY50002; DeeYeebio, Shanghai, China), and protein concentrations were determined using a BCA protein assay kit (DY30204; DeeYeebio, Shanghai, China). Isolated proteins were separated by SDS-PAGE and electro-transferred onto PVDF membranes. Membranes were blocked with commercial blocking solution for 10 min at room temperature with gentle shaking. After blocking, membranes were incubated with primary antibodies overnight at 4 °C, with relevant antibody information provided in Table 1. After washing, membranes were incubated with the HRP-conjugated goat anti-rabbit IgG (1:2000; DY60202, DeeYeebio, Shanghai, China). After further rinsing, protein bands were visualized using a Western Bright ECL chemiluminescence kit (DY30208, DeeYeebio, Shanghai, China), and band images were acquired via the Chemi-Doc XRS imaging system (Bio-Rad, Hercules, CA, USA).

### 2.10. Reverse Transcription-PCR (RT-PCR) Analysis

Total RNA was extracted from cells using TRIzol reagent (9109; TAKARA, Tokyo, Japan) and treated with DNase to remove genomic DNA (gDNA). Complementary DNA (cDNA) was subsequently synthesized using a cDNA synthesis kit (DY90104; DeeYeebio, Shanghai, China). PCR amplification was performed using the cDNA as the template, and the amplified products were separated by electrophoresis on a 2% agarose gel, stained with a nucleic acid dye (DY10214; DeeYeebio, Shanghai, China), and visualized under ultraviolet light. As the negative control, PCR amplification was carried out using total RNA without reverse transcription (–RT) as the template. The primer sequences and expected product sizes for RT-PCR are listed in Table 2.

### 2.11. Quantitative Real-Time PCR (qPCR)

Primary testicular somatic cells and the derived monoclonal SC lines were harvested, and total RNA was directly extracted. Reverse transcription was performed using a cDNA synthesis kit (DY90104; DeeYeebio, Shanghai, China). The obtained cDNA was diluted 30-fold and subjected to detection on an FQD-96A quantitative PCR system (Hangzhou Bioer Technology, Hangzhou, China) using ChamQ Universal SYBR qPCR Master Mix (Q711-02; Vazyme, Nanjing, China) as the reaction system. The thermal cycling parameters were set as follows: initial denaturation at 95 °C for 5 min, followed by 40 cycles of 98 °C for 10 s and 60 °C for 30 s, with a final extension at 72 °C for 10 min. Three independent biological replicates, represented by different cell passages for each cell group, were included in this qPCR assay, and each passage-derived biological sample was assayed in three technical replicate wells. *GAPDH* was adopted as the internal reference gene, and the relative expression levels of target genes were calculated utilizing the 2^−ΔΔCT^ strategy. The primer sequences and qPCR product sizes are detailed in Table 2.

### 2.12. Transmission Electron Microscopy (TEM) Analysis

Immortalized giant panda SC lines grown to confluence with intact tight junctions were harvested. After medium removal, cells were rinsed once with the prewarmed culture medium, gently scraped, and collected via low-speed centrifugation. Cells were resuspended in 2.5% glutaraldehyde electron microscopy fixative, then centrifuged at 10,000 rpm for 10–12 min to form a compact pellet. Following supernatant removal, fresh fixative was added, and samples were incubated at room temperature for 30 min prior to refrigeration at 4 °C. Later, specimens were pre-embedded in 1% agarose, post-fixed with 1% osmium tetroxide for 2 h at room temperature, and rinsed with phosphate buffer. After gradient ethanol dehydration and acetone infiltration, samples were sequentially infiltrated and embedded in 812 embedding resin, then polymerized at 60 °C for 48 h. Ultrathin sections of 60–80 nm thickness were cut using a Leica UC7 ultramicrotome (Leica, Wetzlar, Germany). Sections were double-stained with uranyl acetate and lead citrate, then observed and photographed with a HITACHI HT7800 transmission electron microscope (HITACHI, Tokyo, Japan) at an accelerating voltage of 80.0 kV.

### 2.13. Assessment of SC Responses to Testosterone and Follicle-Stimulating Hormone (FSH)

SCs at the logarithmic growth phase were seeded into 12-well plates at an appropriate density and cultured overnight at 37 °C in a humidified atmosphere containing 5% CO_2_ to allow cell attachment. On the following day, the culture medium was removed and replaced with the fresh complete medium supplemented with either 0 or 10 μM testosterone (T71104; Psaitong, Beijing, China) or 0 or 0.1 U/mL FSH (F8470, Solarbio, Beijing, China). The corresponding vehicle-treated groups were used as controls, with an equivalent volume of dimethyl sulfoxide (DMSO) added to the testosterone control group and sterile physiological saline added to the FSH control group. After 24 h of hormone stimulation, total RNA was extracted using the TRIzol reagent (9109; TAKARA, Tokyo, Japan) according to the manufacturer’s instruction and used for subsequent gene expression analysis. Technical triplicates were applied to each group, and three independent biological experiments were performed.

### 2.14. Karyotyping

Karyotype analysis was conducted on primary testicular somatic cells and immortalized monoclonal SC lines, as previously depicted [31]. Prepared cell slides were examined using a Nikon Eclipse 80i fluorescence microscope (Nikon, Tokyo, Japan). Three independent biological experiments were performed, and 100 cells were counted and analyzed in each group per experiment.

### 2.15. DNA Content Analysis

DNA content of primary testicular somatic cells and immortalized monoclonal SC lines was determined by flow cytometry, as previously reported [31]. Samples were detected with a BD FACS flow cytometer (BD Biosciences, San Jose, CA, USA), and data were analyzed using ModFit LT 32 software version 4.0 (Verity Software House, Topsham, ME, USA).

### 2.16. Statistics

Data were presented as the mean ± standard error of the mean (SEM) of three independent biological experiments. Each experiment was performed using separate culture batches of the corresponding cell line. All experimental data were tested for normality utilizing Kolmogorov–Smirnov and subjected to transformation as necessary to meet the demand of normal distribution. Unless otherwise stated, the two-tailed Student’s *t*-test was used for two-group comparisons, and one-way ANOVA followed by Tukey’s HSD post hoc test was used for multiple comparisons among three groups. Statistical differences were considered significant and highly significant at *p* < 0.05 (*) and *p* < 0.01 (**) or 0.001 (***), respectively.

## 3. Results

### 3.1. Isolation and Immortalization of SC-Enriched Testicular Somatic Cells

**Figure 1:**
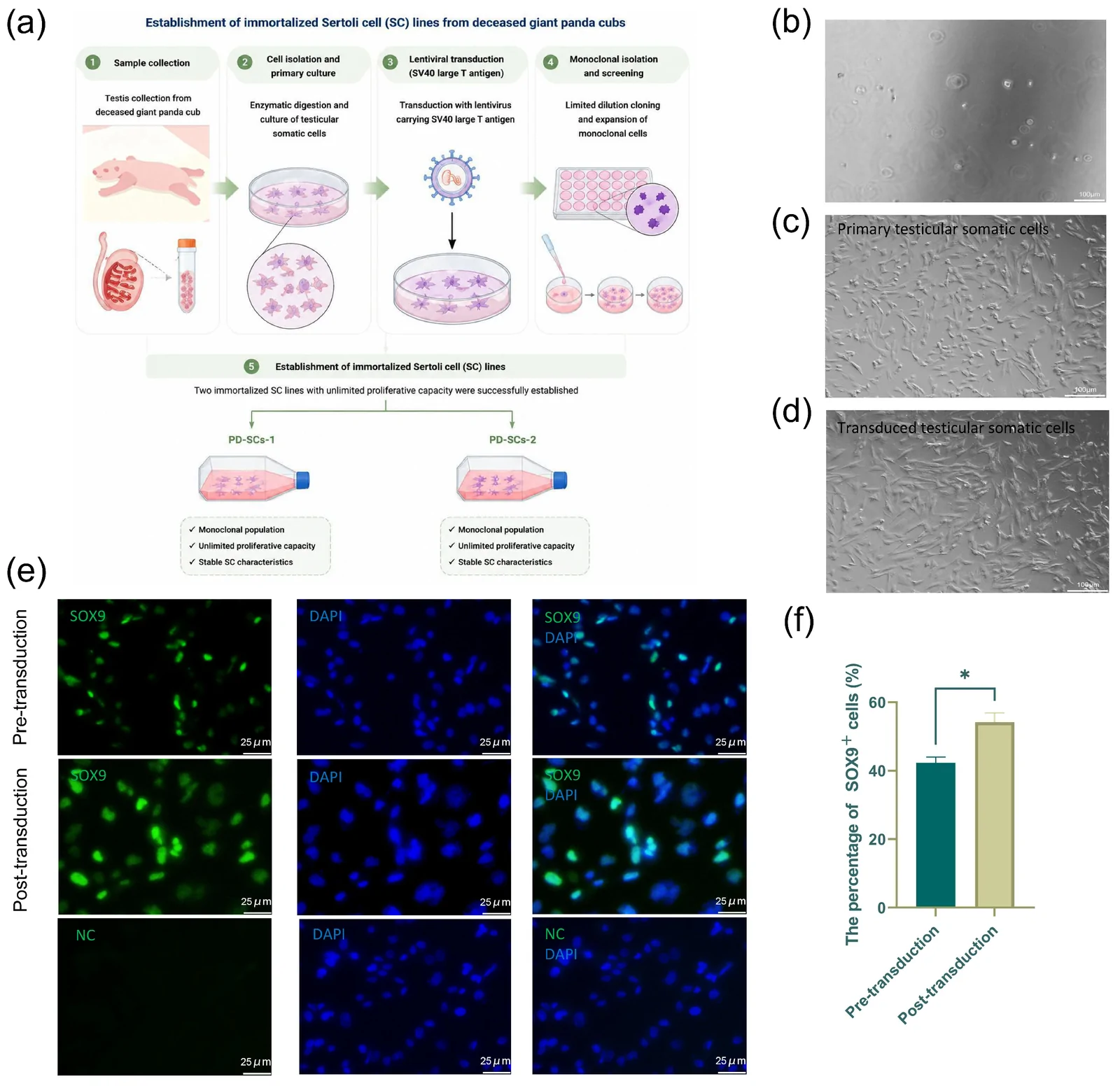
Isolation and immortalization of SC-enriched testicular somatic cells. (**a**) A workflow of establishment of immortalized SC lines from a deceased giant panda cub. (**b**) An image of the single-cell suspension. Scale bar = 100 μm. (**c**,**d**) Images of primary testicular somatic cells before (**c**) and after (**d**) lentiviral transduction. Scale bar = 100 μm. (**e**) Immunofluorescence staining of SOX9 in cells before and after lentiviral transduction. Scale bar = 25 μm. (**f**) Quantification of the proportion of SOX9^+^ cells before and after lentiviral transduction. Data are presented as the mean ± SEM of three independent biological experiments (*n* = 3). *: *p* < 0.05.

This study aimed to establish an immortalized SC line from giant pandas (Figure 1a). Testes were first collected from a deceased giant panda cub, and testicular tissue was then processed using mechanical dissociation combined with a two-step enzymatic digestion method to obtain the single-cell suspension (Figure 1b). After seeding and culture, primary testicular somatic cells were obtained. By passage 3, no obvious germ cells were observed, and the cultured cells maintained a uniform and stable cellular state (Figure 1c). Next, the transfer plasmids encoding the SV40 large T antigen were transduced into primary testicular somatic cells using lentiviruses. No apparent morphological changes were observed after transduction (Figure 1d). Immunofluorescence staining for SOX9 showed that the proportion of SOX9^+^ cells that represent SCs among passage 3 primary testicular somatic cells was 42.3% ± 1.72%, while after transduction and one subsequent passage, the proportion of SOX9^+^ SCs significantly increased to 54.16% ± 2.72% (Figure 1e,f), laying the foundation for subsequent monoclonal isolation and functional characterization of SCs.

### 3.2. Establishment of Monoclonal SC Lines

**Figure 2:**
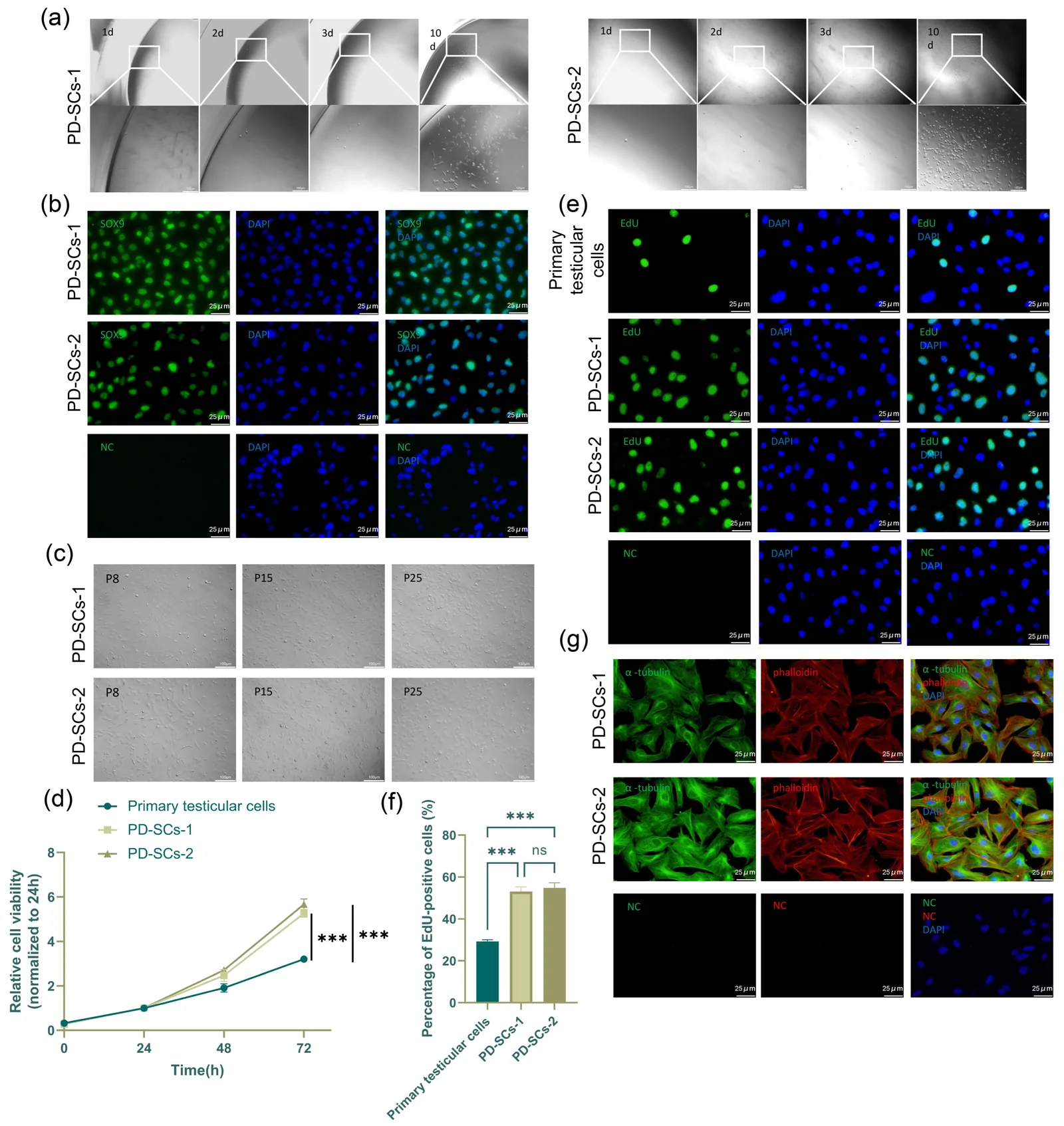
Establishment of monoclonal SC lines. (**a**) Representative images of two monoclonal SC line growth in 96-well plates at 1, 2, 3, and 10 days after seeding. Scale bar = 100 or 25 μm. (**b**) Immunofluorescence staining of SOX9 on two monoclonal cell lines showing complete SOX9 nuclear positivity. Scale bar = 25 μm. (**c**) Representative phase-contrast images of PD-SCs-1 and PD-SCs-2 at passages 8, 15, and 25. Scale bar = 100 μm. (**d**) CCK-8 assay showing the metabolic capacity of primary testicular somatic cells (at passage 4) and PD-SCs-1/2 (at passage 19) at different time points. Data are presented as the mean ± SEM of three independent biological experiments (*n* = 3). ***: *p* < 0.001. (**e**) Representative immunofluorescence images of EdU staining in primary testicular somatic cells (at passage 4) and PD-SCs-1/2 (at passage 20) for cell proliferation detection. Scale bar = 25 μm. (**f**) A statistical histogram showing the quantitative analysis of EdU^+^ cell rates in different cell groups. Data are presented as the mean ± SEM of three independent biological experiments (*n* = 3). ***: *p* < 0.001. (**g**) Co-staining of α-tubulin and phalloidin on PD-SCs-1 and PD-SCs-2. Scale bar = 25 μm.

Because only around half of the immortalized testicular somatic cells were SCs, i.e., the cell population contained other testicular cell types, limiting dilution was subsequently performed using passage 5 immortalized testicular somatic cells, with an aim to establish monoclonal SC lines. After seeding the cells into 96-well plates, wells containing a single cell were marked and monitored daily under a microscope. On day 10 after seeding, 14 monoclonal cell populations were successfully obtained. SOX9 staining showed that only two clones exhibited 100% nuclear SOX9^+^ signals (Figure 2a,b), whereas the remaining clones were only partially positive for SOX9 staining. These two SOX9^+^ monoclonal cell lines were termed PD-SCs-1 and PD-SCs-2, respectively. Visual inspection via light microscopy revealed that both cell lines displayed comparable morphological traits, characterized by an extended, spindle-like or fibroblast-resembling contour accompanied by a notably dense and orderly cellular arrangement. No obvious morphological alterations were observed during continuous passaging (Figure 2c). The CCK-8 assay revealed that PD-SCs-1 and PD-SCs-2 exhibited the similar metabolic activity, markedly higher than that of the original heterogeneous testicular somatic cells (Figure 2d). Consistently, the EdU proliferation assay showed that the proportion of EdU^+^ cells in primary testicular somatic cells was only 29.27% ± 0.83%, while the EdU positivity rates of PD-SCs-1 and PD-SCs-2 both exceeded 50% (Figure 2e,f), in line with the typical vigorous proliferative properties of immortalized cell lines. To evaluate cytoskeletal integrity and long-term stability, PD-SCs-1 and PD-SCs-2 at passage 30 were co-stained for α-tubulin and phalloidin. The results revealed a well-organized cytoskeletal architecture, with no abnormal morphology such as multinucleated cells observed (Figure 2g). To date, PD-SCs-1 and PD-SCs-2 have continuously been maintained in vitro for more than eight months and passaged over 70 times, with no apparent changes in cellular morphology or proliferative capacity.

### 3.3. Characterization of Monoclonal SC Lines

**Figure 3:**
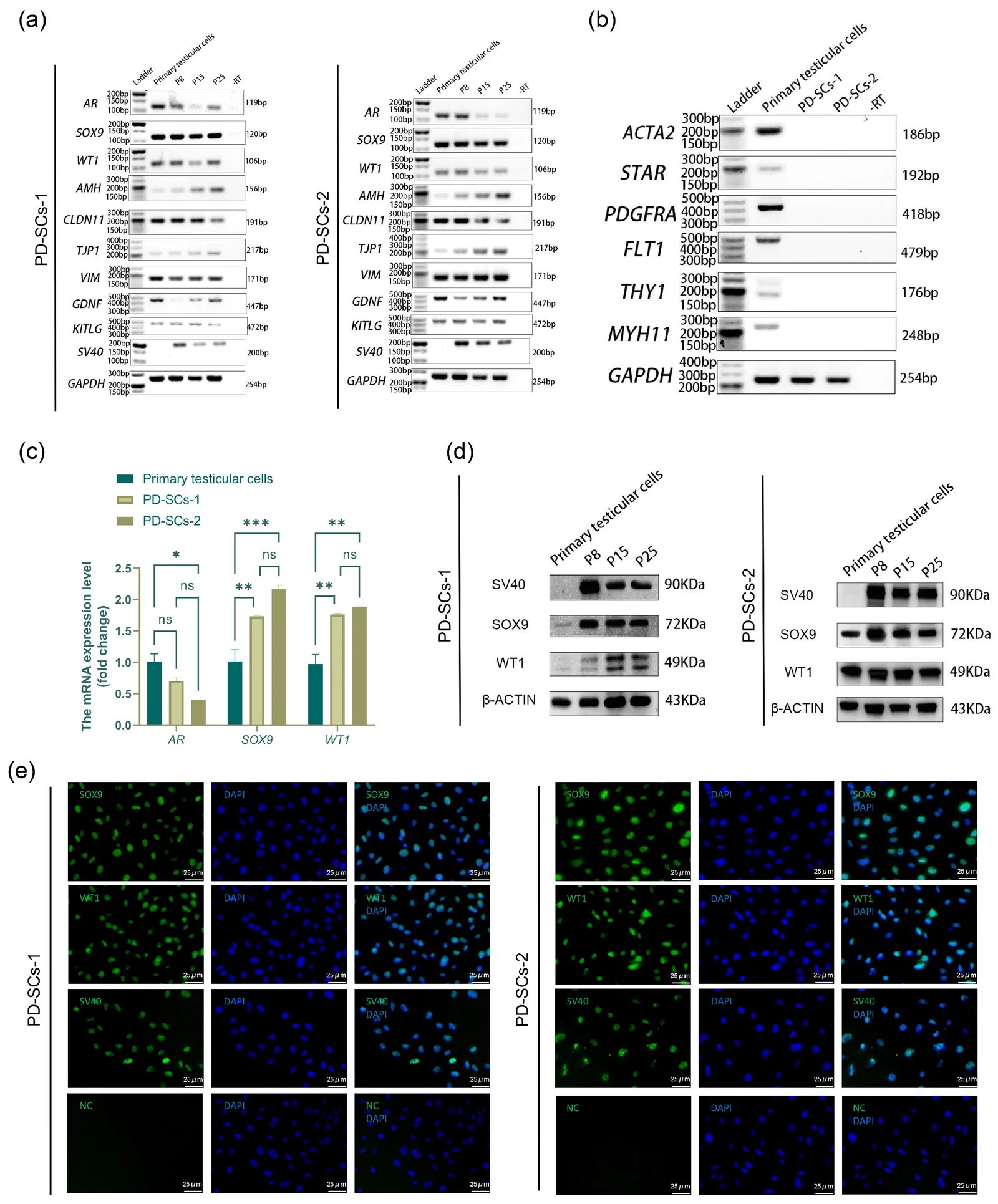
Characterization of monoclonal SC lines. (**a**) RT-PCR analysis showing the mRNA expression levels of *AR*, *SOX9*, *WT1*, *AMH*, *CLDN11*, *TJP1*, *VIM*, *GDNF*, *KITLG*, and *SV40* in primary testicular somatic cells, PD-SCs-1, and PD-SCs-2 at different passages. *GAPDH* was used as the internal reference. –RT denotes the negative control without reverse transcription. (**b**) RT-PCR analysis showing the mRNA expression levels of *ACTA2*, *STAR*, *PDGFRA*, *FLT1*, *THY1*, and *MYH11* in primary testicular somatic cells, PD-SCs-1, and PD-SCs-2 at passage 15. *GAPDH* was used as the internal reference. -RT denotes the negative control without reverse transcription. (**c**) qPCR analysis of *AR*, *SOX9*, and *WT1* expression in primary testicular somatic cells, PD-SCs-1, and PD-SCs-2 at passage 30. Data are presented as the mean ± SEM of three independent biological experiments (*n* = 3). *: *p* < 0.05, **: *p* < 0.01, ***: *p* < 0.001. (**d**) Protein expression levels of SOX9, WT1, and SV40 in primary testicular somatic cells, PD-SCs-1, and PD-SCs-2 at different passages. β-ACTIN was used as the internal reference. (**e**) Immunofluorescence staining of SOX9, WT1, and SV40 on PD-SCs-1 and PD-SCs-2 at passage 35. Scale bar = 25 μm.

To verify the SC identity and immortalized characteristics of the monoclonal cell lines, we performed multidimensional characterization using RT-PCR, Western blot, and immunofluorescence staining. RT-PCR results demonstrated that both PD-SCs-1 and PD-SCs-2 at different passages stably expressed multiple SC markers, i.e., *AR*, *SOX9*, *WT1*, *AMH*, *CLDN11*, *TJP1* (*ZO-1*), *VIM*, *GDNF*, and *KITLG* (*SCF*). Meanwhile, the *SV40* transcript was detected exclusively in the immortalized cell lines but absent in primary testicular somatic cells (Figure 3a). To further validate their SC identity and exclude the contamination of other testicular somatic cells, RT-PCR was performed to detect the expression of markers for peritubular myoid cells (*ACTA2* and *MYH11*), mature (*STAR*) and immature Leydig cells (*PDGFRA* and *THY1*), and endothelial cells (*FLT1*). As expected, these markers were exclusively expressed in primary testicular somatic cells but not in monoclonal cell lines PD-SCs-1 and PD-SCs-2 at passage 15 (Figure 3b). The qPCR analysis further revealed that compared with primary testicular somatic cells, monoclonal cell lines at passage 30 exhibited downregulated mRNA expression of *AR*, whereas the expression levels of *SOX9* and *WT1* showed an upward trend (Figure 3c). Western blot and immunofluorescence staining results were consistent with the transcript-level analysis, showing that PD-SCs-1 and PD-SCs-2 stably expressed the SC markers WT1, SOX9, and the SV40 protein (Figure 3d,e). Collectively, these results indicate that two monoclonal giant panda cell lines expressing SC markers and carrying the SV40 immortalization tag were successfully established.

### 3.4. Functional Validation of Monoclonal SC Lines

**Figure 4:**
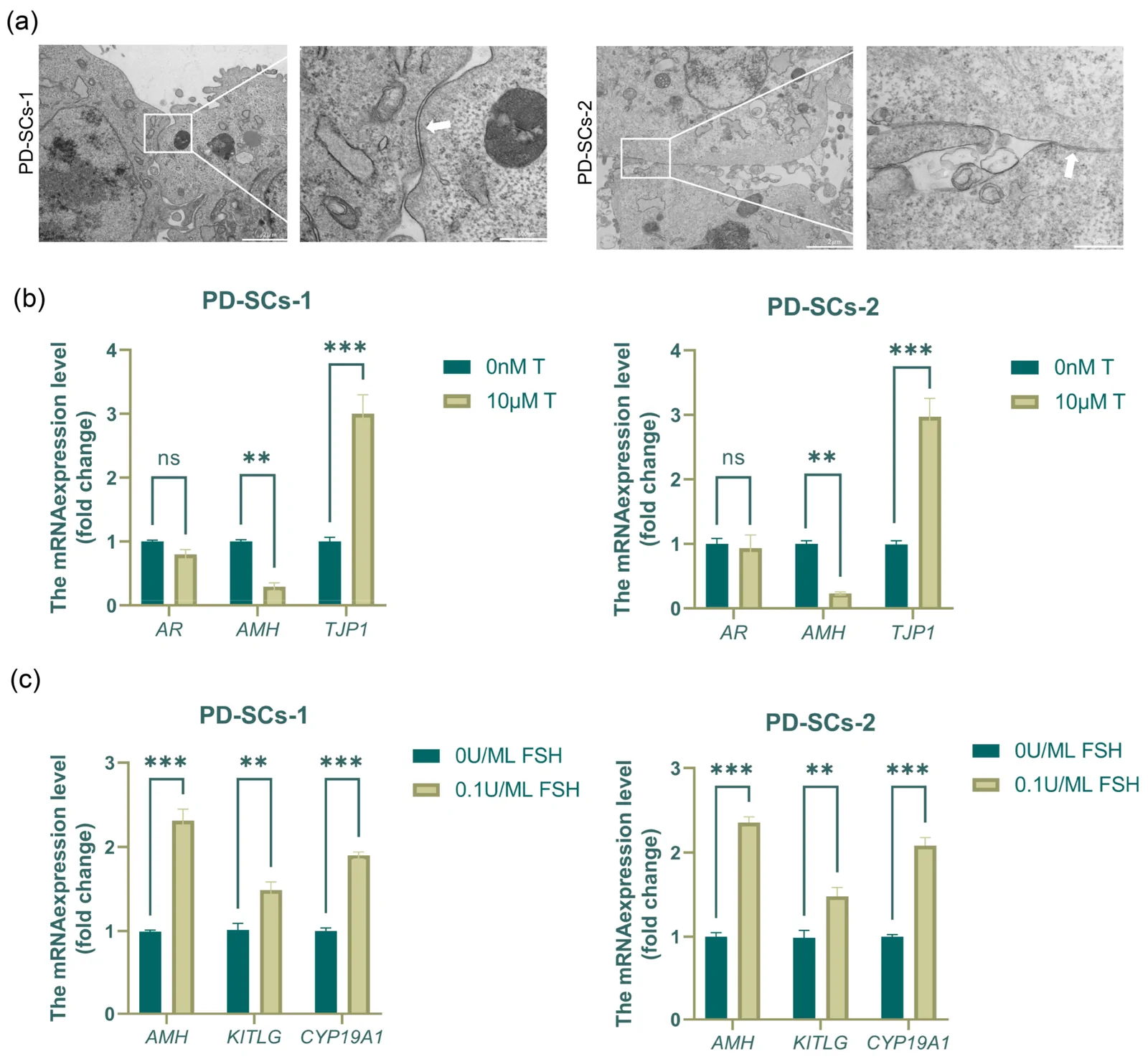
Functional validation of monoclonal SC lines. (**a**) Ultrastructural analysis of PD-SCs-1 and PD-SCs-2 cells by TEM. The left panels show low-magnification images of adjacent cells, and the boxed regions are enlarged in the right panels. Arrowheads indicate regions showing closely apposed plasma membranes between neighboring cells. Scale bars = 2 μm (left panels) or 500 nm (right panels). (**b**) Relative mRNA expression levels of *AR*, *AMH*, and *TJP1* in PD-SCs-1 and PD-SCs-2 cells following testosterone treatment. (**c**) Relative mRNA expression levels of *AMH*, *KITLG*, and *CYP19A1* in PD-SCs-1 and PD-SCs-2 cells following FSH stimulation. Data are presented as the mean ± SEM of three independent biological experiments (*n* = 3). **: *p* < 0.01, ***: *p* < 0.001.

To further evaluate whether the two monoclonal cell lines retain functional characteristics of SCs, the ultrastructural features of intercellular contacts were first examined by TEM. Both PD-SCs-1 and PD-SCs-2 cells were found to exhibit extensive contact with adjacent cells, with variations in the intercellular distance observed among different contact regions. At higher magnification, some areas of cell–cell contacts showed closely apposed plasma membranes with relatively organized membrane-to-membrane alignment (Figure 4a), representing tight junction-like membrane apposition structures. These membrane apposition features suggest that both cell lines retain the morphological characteristics associated with intercellular junction organization.

To further assess whether these monoclonal cell lines retain the hormonal responsiveness characteristic of SCs, their molecular responses to testosterone and FSH stimulation were evaluated. The qPCR analysis showed that treatment with 10 μM testosterone significantly downregulated *AMH* and upregulated *TJP1* at the mRNA level in both PD-SCs-1 and PD-SCs-2 cells (Figure 4b), while stimulation with 0.1 U/mL FSH significantly increased the mRNA expression levels of *AMH*, *KITLG*, and *CYP19A1* in both cell lines (Figure 4c). These results demonstrate that PD-SCs-1 and PD-SCs-2 cells are responsive to androgen and FSH stimulation, supporting the retention of key physiological properties associated with SCs.

### 3.5. Karyotype Analysis of Monoclonal SC Lines

**Figure 5:**
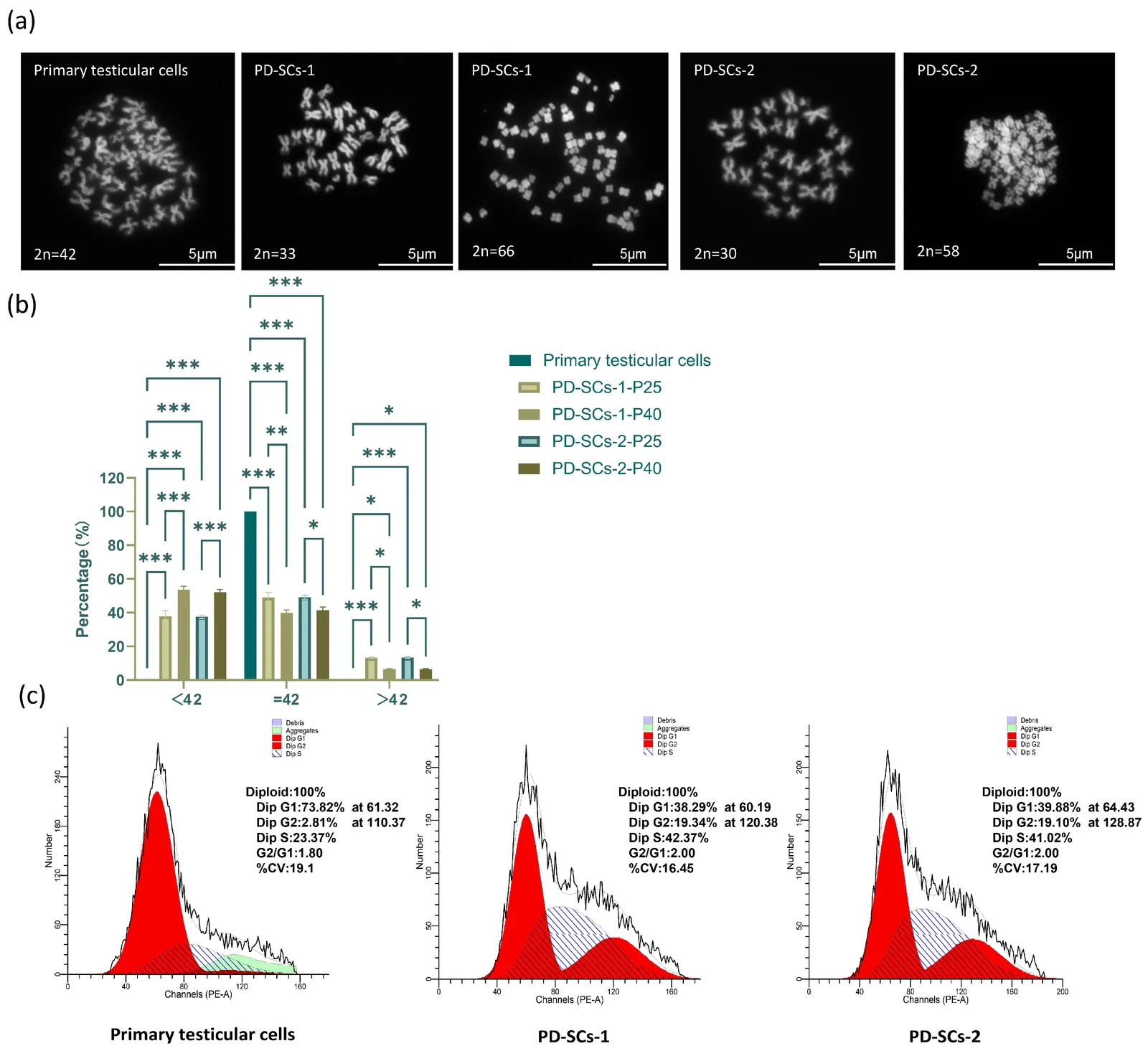
Karyotype analysis of monoclonal SC lines. (**a**) Representative metaphase chromosome images of primary testicular somatic cells, PD-SCs-1, and PD-SCs-2. Scale bar = 5 μm. (**b**) Percentages of cells with a normal karyotype (42 chromosomes) or an abnormal karyotype (> or <42 chromosomes) in primary testicular somatic cells (at passage 5), PD-SCs-1 and PD-SCs-2 (at passages 25 and 40). Data are presented as the mean ± SEM of three independent biological experiments (*n* = 3). *: *p* < 0.05, **: *p* < 0.01, ***: *p* < 0.001. (**c**) DNA content analysis of primary testicular somatic cells, PD-SCs-1, and PD-SCs-2 at passage 28.

Finally, to investigate the effect of SV40 large T antigen-mediated immortalization on genomic stability, karyotype analysis and DNA content assessment were performed on primary testicular somatic cells at passage 5 and PD-SCs-1 and PD-SCs-2 at passages 25–40. Karyotype analysis revealed that primary testicular somatic cells maintained a normal diploid karyotype (2*n* = 42), whereas both immortalized monoclonal cell lines showed varying degrees of chromosomal number abnormalities. Specifically, chromosomal loss was pronounced in both PD-SCs-1 and PD-SCs-2 cells, and the proportion of cells exhibiting chromosomal abnormalities showed an upward trend with the increasing passage number (Figure 5a,b). DNA content analysis further demonstrated that the proportion of cells in S or G2/M phase was markedly higher in PD-SCs-1 and PD-SCs-2 than in primary testicular somatic cells (Figure 5c). These results suggest that SV40 large T antigen-mediated immortalization enables the giant panda SCs to maintain long-term proliferative capacity, which is accompanied by a certain degree of chromosomal instability and altered cell cycle regulation.

## 4. Discussion

SCs are the only somatic cells within the seminiferous tubules of the testis and constitute the core component of the spermatogenic microenvironment. They are involved in nutrient support, BTB formation, and endocrine regulation, and their dysfunction is closely associated with male reproductive disorders [13]. Decades of studies have established AR, SOX9, and WT1 as canonical SC markers, and their expression patterns are known to be stage-specific during development [5]. Currently, SCs remain a research focus in reproductive biology, and the successfully established primary culture and immortalized SC systems in model animals and humans have provided essential tools for elucidation of the regulatory mechanisms for spermatogenesis and development of interventions for male reproductive disorders. Nonetheless, compared with those in model animals, studies on SCs in rare and endangered wildlife species remain scarce. An underlying reason is the absence of suitable cellular models, which severely limits in-depth investigation of their physiological characteristics.

Poor reproductive output among males has long hindered population sustainability in captive giant pandas, an endangered species unique to China, being a critical barrier to their demographic growth. Abnormal SC functions might be one of the important factors underlying the poor reproductive capacity. To date, research on giant panda SCs has been limited to histological observations at the tissue level and transcriptomic analyses at the single-cell level [28]. No stable immortalized SC line with continuous passaging has yet been reported, which has greatly hindered systematic investigations into the regulation of spermatogenesis and the functional characteristics of SCs in this species. To address this gap, here we established two immortalized SC lines, i.e., PD-SCs-1 and PD-SCs-2, from neonatal giant panda testicular tissue through primary isolation and culture, SV40 large T antigen-mediated immortalization, and monoclonal screening. Both cell lines stably expressed SC markers and exhibited some physiological functions of SCs, providing cellular resources with potential applications for future studies on SCs in juvenile giant pandas. More broadly, this work also offers new insights and technical support for fertility preservation in rare and endangered species.

Testicular tissue samples from giant pandas are extremely scarce and typically only available postmortem. In the present study, we obtained a particularly rare juvenile specimen—the testicular tissue of a giant panda neonate that died shortly after birth. Such neonatal samples are exceptionally valuable and rarely available, representing an exceptional resource for biomedical and reproductive research. The karyotypic analysis demonstrated that primary testicular somatic cells isolated from the donor tissue contained 21 pairs of chromosomes, consistent with the normal diploid chromosome number of this species, with no detectable numerical abnormalities or overt structural defects. Notably, because the individual was at an early postnatal developmental stage, the testicular tissue remained in an immature state. In particular, SCs had not yet undergone full terminal differentiation and thus retained relatively high proliferative potential and cellular plasticity, providing favorable biological conditions for in vitro culture and establishment of immortalized cell lines [14].

Building upon this, to overcome the limited proliferative capacity of primary SCs, we further employed an SV40 large T antigen-mediated immortalization strategy. The SV40 large T antigen is one of the most widely used functional proteins in somatic cell immortalization studies. Its primary mechanisms involve binding to tumor suppressor proteins encoded by p53 and Rb, inhibiting their activity, and consequently bypassing cell cycle checkpoints. This enables cells to overcome replicative senescence and achieve long-term stable proliferation in vitro [32]. We employed a lentiviral system to deliver the SV40 large T antigen into testicular somatic cells isolated from the neonatal giant panda, followed by monoclonal isolation and culture. Subsequent experiments validated that the monoclonal cell lines hold long-term proliferative capacity. Specifically, the RT-PCR analysis revealed sustained *SV40* mRNA expression in both PD-SCs-1 and PD-SCs-2, whereas no expression signal was detected in primary testicular somatic cells. Western blotting and immunofluorescence staining further confirmed stable protein expression of the SV40 large T antigen in both cell lines. Additionally, the proliferative capacity of the immortalized cell lines was significantly enhanced compared to that of primary cells. The two cell lines were able to continuously propagate for over eight months, with more than 70 passages, while maintaining stable morphology. These findings collectively demonstrate that the SV40 large T antigen-mediated immortalization is successfully fulfilled in giant panda SCs. Notably, the primary testicular somatic cell population used in this study was heterogeneous, with SOX9^+^ cells accounting for approximately 42% of the total population, and because 100% pure primary SCs could not be obtained due to technical limitations, PD-SCs-1 and PD-SCs-2, two SOX9^+^ monoclonal cell lines, were obtained through screening. Therefore, the differences in gene expression and cell proliferation between the primary cell population and PD-SCs-1/2 may reflect not only the effects of immortalization but also substantial differences in cellular composition. In this sense, the present study demonstrates the acquisition of long-term proliferative capacity during cell line establishment, rather than directly quantifying the specific effects of SV40 on primary SCs. Future studies using highly enriched primary SCs from multiple donors would be necessary to more precisely disentangle the effects of development, cellular composition, and immortalization.

Two cell populations were isolated from neonatal giant panda testicular cells through monoclonal screening, and all examined cells showed positive nuclear staining for SOX9. No expression of markers associated with peritubular myoid cells, Leydig cells, or endothelial cells was detected in the established cell clones, thereby supporting their SC identity and cellular purity. WT1 and SOX9 were stably expressed at both the transcription and protein levels during passaging, while other SC markers were also consistently detected at the mRNA level. These findings lend further support to the retention of recognizable SC-related phenotypes in both clones and indicate the successful establishment of two SC lines from the neonatal giant panda. As these cells were derived from a neonatal giant panda that died shortly after birth due to developmental abnormalities, potential effects of genetic, developmental, or systemic factors on the cellular characteristics cannot be excluded. In addition, neonatal SCs are at a stage of active proliferation and progressive maturation and therefore differ from mature adult SCs in terms of proliferative status, differentiation, and related functional characteristics [33]. Consequently, PD-SCs-1 and PD-SCs-2 should primarily be regarded as cell lines derived from neonatal giant panda SCs rather than direct substitutes for the physiological functions of adult SCs. Also, the findings of the present study should not be generalized to SCs from other individuals or developmental stages of giant pandas.

For SCs to serve as a dependable in vitro experimental platform, both their operational competence and chromosomal soundness must be rigorously appraised. Hence, we assessed these attributes using several complementary techniques. TEM revealed that PD-SCs-1 and PD-SCs-2 retained SC-related intercellular junctional structures, including some structures resembling tight junctions. This finding is consistent with the developmental characteristics of SCs, that the assembly of a functional BTB depends on pubertal hormonal signals and interactions with germ cells, and that fully functional tight junctions are generally established progressively after puberty [34,35]. Nevertheless, direct measurements of barrier permeability or transepithelial electrical resistance were not performed in the present study. Also, owing to the absence of commercially available antibodies suitable for giant pandas, protein-level localization analyses of key BTB components, such as CLDN11 and ZO-1, were not performed. Future studies combining the localization and quantitative assessment of tight junction proteins with direct barrier function measurements are needed to unravel whether these cells can recapitulate the functional barrier properties of SCs.

As shown by the qPCR results described above, the expression of *AR* was significantly lower in the monoclonal cell lines than in the primary testicular somatic cell population. As a matter of fact, reduced *AR* expression has also been reported in some SV40-mediated immortalized SC models, which may be associated with alterations in regulatory states induced by immortalization [36]. However, because AR is a key regulator of SC maturation and androgen-responsive functions, its reduced expression may substantially affect the suitability of these cell lines for studies specifically focusing on androgen-dependent SC biology. To further evaluate this limitation, we examined the responses of PD-SCs-1 and PD-SCs-2 to testosterone and FSH. Testosterone treatment significantly decreased *AMH* expression while increasing *TJP1* expression in both PD-SCs-1 and PD-SCs-2, indicating that despite the reduced AR expression, both cell lines retain detectable responsiveness to testosterone. FSH stimulation, in turn, increased the expression of *AMH*, *KITLG*, and *CYP19A1* in both cell lines. These coordinated transcriptional responses indicate that the established cell lines remain responsive to androgen- and FSH-related stimulation. Nevertheless, these results should be considered as evidence of hormone responsiveness at the molecular level rather than the proof that all androgen- or FSH-dependent physiological functions of mature SCs have been retained. Further analyses at the protein level would be needed to more profoundly characterize the extent and functional relevance of hormone responsiveness in these cell lines.

Karyotype and cell cycle analyses showed that the primary testicular somatic cells retained the normal diploid chromosome number of 42, whereas both PD-SCs-1 and PD-SCs-2 exhibited marked numerical chromosomal abnormalities, including chromosome loss. Moreover, the proportion of cells with abnormal chromosome numbers showed an upward trend with the increasing passage number. The DNA content analysis further revealed alterations in cell cycle distribution, with the immortalized cell lines showing significantly increased proportions of cells in the S and G2/M phases after overcoming the senescence-associated proliferative limitation, consistent with enhanced proliferative activity. These findings indicate that the acquisition of long-term proliferative capacity is accompanied by alterations in the chromosome number and cell cycle regulation. Although genomic abnormalities are common during oncogene-mediated immortalization and may be mechanistically associated with disruption of p53/Rb-dependent cell cycle regulation, and similar phenomena have been reported in various immortalized cell lines [37,38], the abnormalities observed in the present study should not be regarded simply as an expected consequence of SV40 expression or biologically negligible changes. Rather, these abnormalities represent an important characteristic of the established cell lines and may influence gene expression, cellular signaling, endocrine responsiveness, and long-term functional properties.

Therefore, it is important to distinguish long-term culture stability from genomic stability when considering the characteristics and potential applications of these cell lines. The absence of obvious morphological changes after more than 70 passages indicates sustained adaptation to in vitro culture conditions but not necessarily the maintenance of a normal or stable karyotype. The observed chromosomal abnormalities, particularly their increasing frequency with the increasing passage number, suggest that genomic stability may progressively decline during prolonged culture. Accordingly, PD-SCs-1 and PD-SCs-2 may have potential utility for investigation of SC-related molecular responses, signaling pathways, and cellular processes that are less dependent on the normal chromosomal state. However, caution is warranted when these cell lines are used in studies requiring faithful representation of normal SC physiology, chromosome-dependent phenotypes, or long-term genomic fidelity. Monitoring chromosomal status at defined passage numbers and, where appropriate, using early-passage cells may help minimize passage-related variations and improve the reproducibility of future applications.

To sum up, this study established two monoclonal cell lines, PD-SCs-1 and PD-SCs-2, from neonatal giant panda testicular tissue, both of which exhibited long-term proliferative capacity and retained several molecular and functional characteristics associated with SCs. Both cell lines were confirmed free of mycoplasma contamination and their identity was verified by karyotypic analysis and marker-based characterization. On the other hand, reduced *AR* expression, numerical chromosomal abnormalities, and the neonatal origin from a single donor highlight salient limitations of these cell lines as experimental models. Therefore, PD-SCs-1 and PD-SCs-2 should be regarded as experimental resources for investigation of SC-related molecular regulation and cellular processes in juvenile giant pandas, rather than complete substitutes for normal adult SCs. Despite the limitations, these cell lines provide valuable experimental materials for studies on giant panda SCs that are otherwise difficult to obtain, and their use may facilitate further investigation of SC functions during spermatogenesis and provide insights into the cellular and molecular bases of reproductive disorders in giant pandas. At present, the cell lines are not deposited in a public cell bank but can be made available to other researchers upon reasonable request and approvement by the relevant administrative department in Chengdu Research Base of Giant Panda Breeding. Future studies using SC lines derived from multiple individuals and different developmental stages, together with broader molecular profiling, protein-level characterization, and functional barrier assays, would help further define the biological fidelity and application scope of these cell lines and promote their appropriate use in giant panda reproductive conservation research. Such efforts are expected to lay a more solid scientific foundation for fertility preservation and contribute to the health and sustainable management of captive giant panda populations and other rare and endangered species.

## Figures and Tables

**Table 1:** Antibody information.

Antibody	Species Source	Supplier	Identifier	Dilution (IF)	Dilution (WB)
SOX9	Rabbit	Abcam	ab185966	1: 200	1: 5000
WT1	Rabbit	HUABIO	ET1610-45	1: 200	1: 2000
SV40	Rabbit	Cell Signaling Technology	15729S	1: 200	1: 1000
α-tubulin	Rabbit	Proteintech	11224-1-AP	1: 200	
Phalloidin		Yeasen Biotechnology	40734ES75	1: 200	
β-actin	Rabbit	Proteintech	81115-1-RR		1: 5000

**Table 2:** (q)RT-PCR primer and product information.

Gene	Primer Sequence (5′-3′)	Product Size (bp)	Accession Number
*SV40*	F: TTTGGAGGCTTCTGGGATGC	200	
	R: CAGTAGCAATCAACCCACACAAG		
*GAPDH*	F: GTGATGCTGGTGCTGAGTATGT	254	XM_001304846.1
	R: CTCCACGATGCCGAAGTTG		
*SOX9*	F: ACGGAGCAGACACACATCTC	120	XM_019804528.2
	R: CTGGGATTGTCCCGAGTGC		
*WT1*	F: CACCAGGACTCATACAGGTGA	106	XM_034646290.1
	R: CTGGTGCATGTTGTGATGGC		
*AMH*	F: GACCTGGGAGCCAACATTCT	156	XM_034657421.1
	R: GACCAGCAAAGGTTCTGGGTG		
*CLDN11*	F: GTGGGTTGGATCGGCATCA	191	XM_002920835.4
	R: TGGACATAGCCCGGCAAG		
*TJP1*	F: ACTCCTCGTCGGGTGATCCT	217	XM_034667960.1
	R: GGTAAGGCATTCCTGCTGGTT		
*VIM*	F: AGAGACGGGCAGGTTATCAA	171	XM_002918630.4
	R: ATCTAGCTTGCGCTCCTGAA		
*GDNF*	F: TTGGACGTCGGTTTCTGGAG	447	XM_034656996.1
	R: CTGGCATGTTTGAGTCACGG		
*KITLG*	F: GGTCCTGAAAAAGGGAAGGC	472	XM_019802248.2
	R: GAGTCCTGCTCCATGCAAGT		
*ACTA2*	F: TCACCAACTGGGACGACAT	186	XM_011218625.3
	R: CCTGGATAGCCACATACATAGC		
*STAR*	F: GCCACACGCTTCGGAGAGAT	192	XM_002917230.4
	R: GTCCACCTGGGTCTGGGATAAG		
*PDGFRA*	F: CCCGGACAGTATAAGAAGAGTTACG	418	XM_002919370.4
	R: AATCTGCCAGTCACAGGAAAC		
*FLT1*	F: ACCGAACAACGCATCACAAC	479	XM_011233822.3
	R: GGACTTGTCTGAGGTTCCTTGAA		
*THY1*	F: CTCCCACCCCTGGTGAAAACT	176	XM_019793142.2
	R: CGGGCTACCTGCAAGACTGT		
*MYH11*		248	XM_034670325.1 XM_034670324.1 XM_034670323.1 XM_034670322.1
	F: GCAATGCCAAAACGGTCA		
	R: GGAGAGGAATGTGTAGTTGTTGAAG		

*CYP19A1*	F: AGGAGGTCAGCAACGAACTG	348	XM_002920421.4
	R: TCAGGTCACCACGTTTCTCG		
*AR*	F: CTATGGGGACATGCGTTTGGA	118	XM_002929171.4
	R: TCCATAGTGACAGCCAGAAGC		

## Data Availability

All data are included in the article.
